# PASS: Protein Annotation Surveillance Site for Protein Annotation Using Homologous Clusters, NLP, and Sequence Similarity Networks

**DOI:** 10.3389/fbinf.2021.749008

**Published:** 2021-09-29

**Authors:** Jin Tao, Kelly A. Brayton, Shira L. Broschat

**Affiliations:** ^1^ School of Electrical Engineering and Computer Science, Washington State University, Pullman, WA, United States; ^2^ Department of Veterinary Microbiology and Pathology, Washington State University, Pullman, WA, United States; ^3^ Paul G. Allen School for Global Health, Washington State University, Pullman, WA, United States

**Keywords:** protein annotation, machine learning, natural language processing, homologous clusters, network science, web application

## Abstract

Advances in genome sequencing have accelerated the growth of sequenced genomes but at a cost in the quality of genome annotation. At the same time, computational analysis is widely used for protein annotation, but a dearth of experimental verification has contributed to inaccurate annotation as well as to annotation error propagation. Thus, a tool to help life scientists with accurate protein annotation would be useful. In this work we describe a website we have developed, the Protein Annotation Surveillance Site (PASS), which provides such a tool. This website consists of three major components: a database of homologous clusters of more than eight million protein sequences deduced from the representative genomes of bacteria, archaea, eukarya, and viruses, together with sequence information; a machine-learning software tool which periodically queries the UniprotKB database to determine whether protein function has been experimentally verified; and a query-able webpage where the FASTA headers of sequences from the cluster best matching an input sequence are returned. The user can choose from these sequences to create a sequence similarity network to assist in annotation or else use their expert knowledge to choose an annotation from the cluster sequences. Illustrations demonstrating use of this website are presented.

## 1 Introduction

Recent advances in the development of high-throughput sequencing technologies and computing capacity have greatly improved the speed of genome sequencing and, as a consequence, have contributed to the exponential growth of genomes in public repositories (Tao et al., 2021). Concomitantly, computational analysis, homology-based inference, and prediction are being widely used to annotate proteins, but the majority of annotations have not been experimentally verified. Lack of experimental verification contributes to inaccurate protein annotation and the propagation of existing annotation errors. The inaccuracy and confusion associated with protein annotation has been well reported, and it is well known that numerous protein sequences are missing annotation (Galperin and Koonin, 1998; Gilks et al., 2005; Schnoes et al., 2009; Salzberg, 2019; Lockwood et al., 2019; Tao et al., 2021). For example, in (Lockwood et al., 2019) homologous clustering was performed using protein sequences downloaded from the National Center for Biotechnology Information (NCBI) GenBank ftp service (Benson et al., 2005), and it was found that among 2,826 Proteobacterial protein sequences within one homologous cluster, 82% of the sequences used GroEL or GroL in their annotation while annotations of the remaining sequences included “not yet annotated,” *mopA* (obsolete gene name), thermosome, and 60 kDa chaperonin (11.78%). Moreover, 44 non-GroEL protein sequences were incorrectly annotated as chaperonin GroEL by the authors submitting the sequences. For this universally conserved protein, the preferred annotation in the UniProtKB/Swiss-Prot database is 60 kDa chaperonin while NCBI RefSeq annotates it as molecular chaperone GroEL (Lockwood et al., 2019), demonstrating a clear inconsistency even between the two databases. In (Mohanta et al., 2020), an analysis performed on 7.15 million protein sequences of 689 fungal species found that the protein sequence annotated as “calcium dependent protein kinase” did not, in fact, encode calcium binding EF-hands in the regulatory domain. In addition, protein sequences with annotations containing “selenocysteine” did not encode the Selenocysteine (U) amino acid. The latter work demonstrates that protein misannotation is not only a problem for bacterial species, but it is also a problem for fungi. The awareness of problems with protein annotation and, in particular, the issue of error propagation, indicates that a tool to help life scientists with accurate protein annotation would be useful.

Traditional methods for protein annotation involve mass spectrometry, microscopy, and RNA interference. Unfortunately, these methods are time-consuming and require the use of substantial resources due to their low throughput and restricted scope of methodology (Schnoes et al., 2013; Cao and Cheng, 2016; Frasca and Cesa-Bianchi, 2017; Hong et al., 2020). As a result, automatic methods for annotation have been adopted, and in recent years, considerable work on the automatic annotation of proteins has been performed. In (Fleischmann et al., 1999), the annotation algorithm extracts conditions from an external database, groups the UniProtKB/Swiss-Prot database entries that fulfill each condition to find the common annotation among each group, and finally extends the annotation to entries from the uncurated UniProtKB/TrEMBL database that are grouped by the same conditions. However, this method has a low coverage rate of 10% and focuses on high specificity because it is impractical to enumerate all the rules and conditions. In (Steinegger et al., 2019), the method used vectorizes and accelerates profile Hidden Markov Model (HMM) alignment which extends sequence profiles by increasing position-specific amino acid substitution scores with position-specific penalties for insertions and deletions. The idea is to use homology detection for deep functional annotation. However, protein homology does not guarantee the same molecular function, and further annotation verification and evaluation are essential. In (Xie et al., 2002), the authors achieve protein annotation based on sequence homology with Gene-Ontology-annotated proteins and protein domain analysis. Though text information is used to improve annotation accuracy, the relationships between specific words and specific Gene Ontology (GO) are based on term frequency only, ignoring semantic information. While cutoff thresholds can be made more stringent in traditional similarity-based methods such as HMMER (Finn et al., 2011) to improve the accuracy of homology detection, recent efforts in automatic protein annotation have turned to machine learning (ML) techniques. ML methods use features that are correlated with a specific function in a training set containing experimentally annotated proteins, formulating annotation as a multi-class classification problem using models such as support vector machines and deep neural networks (Radivojac et al., 2013; Fa et al., 2018; Sun et al., 2020; Zhang et al., 2020). For example, (Nakano et al., 2019) explores and evaluates the ability of hierarchical multi-label classification methods to detect missing or incorrect annotations in Functional Catalogue (FunCat) or GO benchmark datasets. In (Hong et al., 2020), the authors consider the effectiveness of applying a convolutional neural network (CNN) method together with a protein encoding strategy to improve prediction stability. However, none of these efforts in annotation prediction use reliable verification and validation of protein annotations based on experimental evidence.

In this paper, we consider an alternative approach to accurate protein annotation. We introduce the Protein Annotation Surveillance Site (PASS), which provides a tool for life scientists to choose accurate annotations for protein sequences. This website has three major components: a database of homologous clusters of protein sequences representing the four domains bacteria, archaea, eukarya, and viruses, together with sequence information; a machine-learning software tool which periodically queries the UniprotKB database to determine whether protein function has been experimentally verified; and a query-able webpage where the FASTA headers of sequences from the cluster best matching an input sequence are returned. Protein sequences that have been marked by the human curator as having been verified or have “function” publications in the Swiss-Prot database are identified by colored FASTA text. Because homology does not guarantee the same molecular function, the user can use their expert knowledge to choose an annotation from the cluster sequences or else they can create a sequence similarity network (SSN) to assist with annotation. The SSN indicates the sequences most closely related to their query sequence. A sequence that has been confirmed experimentally is listed in color with the SSN results.

A major advantage of using our alternative approach to annotation, e.g., compared to using BLASTp, is that sequences matching the query sequence are provided in the form of a homologous cluster of sequences representing multiple species, potentially from four different domains. Moreover, our database contains more than 360,000 homologous clusters containing more than eight million sequences. Illustrations demonstrating use of the PASS website are given in the Results and Discussion section.

## 2 Materials and Methods

Our annotation pipeline contains three major components: homologous clusters of close to 8.5 million protein sequences deduced from the representative genomes of bacteria, archaea, eukarya, and viruses, which contribute to a database containing all the sequence information; a machine-learning software tool which periodically queries the UniprotKB database to determine whether protein function has been experimentally verified; and a query-able webpage where the FASTA headers of sequences from the cluster best matching an input sequence are returned. Each component is addressed in the following subsections.

### 2.1 Large-Scale Homologous Clustering Using pClust and Sub-Cluster Merging

We collected the protein sequences for our database using the Genome Information Browser by Organism available through the National Center for Biotechnology Information (NCBI) in July 2020, choosing only organisms with completely sequenced genomes in the representative category of RefSeq. The average sequence length is 350 amino acids while the longest length is 36,805. Table 1 shows the number of organisms we collected in each domain.

**TABLE 1:** Number of representative genomes of bacteria, archaea, eukarya, and viruses obtained using the Genome Information Browser by Organism available through the National Center for Biotechnology Information (NCBI) in July 2020.

Bacteria	Eukarya	Virus	Archaea
2,989	16^a^	47	201

a Of the 39 entries meeting the requirements, 23 did not have RefSeq links at the time of collection.

The total number of protein sequences for the 3,253 organisms was approximately 11.2 million including duplicate sequences and sequences with the annotation “hypothetical protein.”

#### 2.1.1 Homologous Clustering Using pClust

There are numerous methods for clustering protein and gene sequences, but here we mention only a few. One novel approach by (Abnousi et al., 2018) uses an alignment-free technique to cluster protein sequences. Another novel approach uses a 3-dimensional method to cluster gene expression data (Lambrou et al., 2019). For our work, we used the pClust method, in part because it worked well for our iterative approach. To generate our homologous protein clusters, we applied the multi-core version of the pClust pipeline (Lockwood, 2016), employing semi-global pairwise sequence alignment with Parasail (Daily, 2016) and scalable community detection with Grappolo (Lu et al., 2015). First, Parasail generates an undirected graph with edges connecting similar sequences, where the sequences are the vertices of the graph. Grappolo then uses the graph results from Parasail to create clusters.

Parasail (Daily, 2016) stands for Pairwise Sequence Alignment Library, and it is a SIMD C library which includes implementations of the Smith-Waterman local sequence alignment, Needleman-Wunsch global sequence alignment, and semi-global sequence alignment algorithms. For this work, we used the semi-global pairwise sequence alignment algorithm because we did not want to penalize gaps at the beginning and/or end of an alignment when the downstream part of one sequence overlapped with the upstream part of the other or when the lengths of two aligned sequences differed significantly. The input for Parasail is a set of protein sequences, and the output is an undirected graph with three alignment statistics for each edge computed, where an edge indicates sequence similarity between two protein sequences. The three alignment statistics are length of alignment over maximum length, number of exact matches over alignment length, and alignment score over self score. We focused on the first alignment statistic, using it as the weight for each edge. It represents the minimum required length of alignment relative to the longest sequence among all sequences to be aligned, and the default threshold is 80%, which has been shown to give optimal results (Khaledian and Broschat, 2020). Only edges with values greater than this threshold were kept to ensure strong similarity between sequences.

Grappolo (Lu et al., 2015) clusters the sequences by parallelization of the Louvain heuristic for community detection in large-scale graphs. Community detection is an NP-complete problem (Brandes et al., 2007) requiring a brute force approach to solve. The Louvain method (Bader et al., 2012), an iterative and greedy heuristic algorithm for producing hierarchical communities, was proposed as an efficient method for solving the community detection problem. Initially, each vertex, or protein sequence, exists in a separate community. Closely related protein sequences are considered members of one community. Modularity (Newman and Girvan, 2004) is defined as the fraction of edges existing within communities minus the expected fraction of edges if they were distributed randomly. It measures the quality of a particular division of a network, and an increase in modularity, representing an improvement in the quality of a network partition, is called its gain. Modularity gain is computed after each iteration until a desired threshold of 10^–6^ is reached. This threshold balances speed of convergence and the quality of the final network partition (Lu et al., 2015). As mentioned, Grappolo is a parallelization of the Louvain heuristic. In each iteration of Grappolo, all the vertices are scanned in parallel in an arbitrary but predetermined order. For a given vertex, the communities containing its neighbors are examined and the modularity gain is computed when the vertex is moved from its current community to each of the neighboring communities. The input to Grappolo is an undirected weighted graph, and the output is a partitioning of communities, or clusters, containing a subset of vertices (protein sequences) for which modularity has been maximized. It should be noted that a community can consist of a single vertex which is called a singleton.

#### 2.1.2 A Two-Step Iterative Clustering and Sub-Cluster Merging Scheme

The ∼11.2 million protein sequences for the 3,253 organisms were downloaded in FASTA files. The sequences were divided into batches, and the sequences annotated as hypothetical proteins as well as duplicate sequences were removed from each batch; ∼9.26 million sequences remained. This number included more than 191,000 duplicate sequences that we were unable to remove because they were distributed across different batches. Some of these duplicates were removed later.

Given the constraints of the computing facilities available to us, it was impossible to cluster the ∼9.26 million sequences as a single set. Because of this, we considered clustering each batch and then combining clusters and singletons from each batch. However, this posed several challenges, including how to name each cluster and singleton in different batches to avoid confusion and how to combine them to achieve accurate results.

We decided that a suitable approach for determining homologous clusters was to adopt a two-step scheme using iterative clustering with sub-cluster merging. To implement our approach, we start by dividing all protein sequences into batches and clustering each batch independently, obtaining homologous clusters and singletons for each batch. Because the homologous clusters are not in their final form, we use the term sub-clusters to describe them. In the next iteration, we reduce the number of sequences and batches by selecting one representative sequence from each sub-cluster (to enable faster sequence-cluster mapping, we select the first sequence within each sub-cluster as the representative), adding all the representative sequences and singletons from a batch to form a new batch, combining several new batches, and clustering the sequences in these. In the final iteration, clustering is performed on a single batch. Sub-clusters generated in the current iteration indicate how sub-clusters and singletons generated in the previous iteration are merged. For example, if two sequences representing sub-clusters in two different batches in the previous iteration cluster together in the current iteration, the two sub-clusters in the previous iteration will be merged. Sub-cluster and singleton merging starts with the results of the final iteration, and conceptually, sequences within each sub-cluster from the final batch are mapped to and merged with sub-clusters or singletons generated in the previous iteration. This is performed repeatedly until sub-clusters and singletons obtained during the first iteration have been merged appropriately. Details on the two steps of our scheme are described in the following sub-sections.

#### 2.1.3 Iterative Clustering

For the first iteration, we started with the ∼9.26 million sequences distributed in eleven batches. The first ten batches consisted only of bacterial sequences, while the 11th batch contained sequences from bacteria, archaea, eukarya, and viruses. To avoid confusion between sequences in different batches, we adopted the following naming convention: We used the uppercase letters A-K to designate the eleven separate batches followed by a number to indicate the index of the sub-cluster, for example, A_0 (indexing begins at 0) designates the first sub-cluster in the first batch and K_9 the 10th sub-cluster in the 11th batch. Individual sequences within a batch were labeled by the lowercase equivalent of the batch letter followed by an index value. This sequence index is determined by pClust which indexes each sequence in a single batch sequentially from start to finish. To identify a sequence in a sub-cluster, we simply concatenate the sub-cluster name and the sequence label for a given batch, for example, A_11#a_15 means the sequence labeled a_15 from batch 1 is in the 12th sub-cluster in batch 1. For singletons, we prefix the original sequence label by S and concatenate this with the original sequence label, for example, for a sequence labeled a_1 in batch 1, Sa_1#a_1 means the second sequence in batch 1 is a singleton and Sa_1091#a_1091 means the 1092nd sequence in batch 1 is a singleton.

In our first iteration, we used pClust to cluster each of the eleven batches of sequences separately. The number of input sequences used and the number of sub-clusters and singletons obtained for each batch are presented in Table 2. Also given in this table are the number of output sequences obtained from the first iteration for use in the second iteration. For each batch, we combined all the singletons and the first sequence from each sub-cluster to represent it, producing a new and much smaller set of input sequences designated as “Output Seqs” in Table 2. The ratio in the last row and column of this table shows a reduction in overall input size for the second iteration of almost 80%. Note that after the first iteration, singletons can combine with other singletons to form sub-clusters in the next iteration or they can combine with other sub-clusters.

**TABLE 2:** “Input Seqs” denotes the number of input sequences for each batch in the first iteration; “Sub-clusters” denotes the number of sub-clusters obtained from each batch; “Singletons” denotes the number of singletons obtained for each batch; “Output Seqs” gives the number of output sequences from the first iteration to be used in the second iteration; and “Out/In” indicates the reduction in size of the sequences to be used in the second iteration compared to the first. The reduction in input size for the second iteration is almost 80%. Each value in the “Output Seqs” column is the sum of the number of sub-clusters and the number of singletons. The first sequence in each sub-cluster was used to represent the entire sub-cluster in the second iteration.

Iter 1	Input seqs	Sub-clusters	Singletons	Output seqs	Out/In (%)
Batch 1	788,410	61,842	138,042	199,884	25.35
Batch 2	820,250	62,774	165,842	228,616	27.87
Batch 3	860,911	68,321	156,397	224,718	26.10
Batch 4	886,836	63,252	120,205	183,457	20.69
Batch 5	898,559	63,882	123,095	186,977	20.81
Batch 6	977,601	66,695	122,559	189,254	19.36
Batch 7	940,736	69,612	122,955	192,567	20.47
Batch 8	918,110	67,816	132,898	200,714	21.86
Batch 9	945,086	67,356	135,681	203,037	21.48
Batch 10	769,210	49,596	89,756	139,352	18.12
Batch 11	451,428	33,248	64,939	98,187	21.75
Total	9,257,137	674,394	1,372,369	2,046,763	22.11

For the second iteration, we combined the output sequences from batches 1, 2, and 3 together, the output sequences from batches 4, 5, 6, and 7 together, and the output sequences from batches 8, 9, 10, and 11 together to form three new batches of sequences. We used pClust on each of these three batches to obtain sub-clusters. The number of input sequences used and the number of sub-clusters and singletons obtained are given in Table 3. Again, the number of output sequences in this table is the sum of the number of singletons and the number of sub-clusters, and the ratio in the last row and column shows a reduction in input size for the third iteration of almost 30%.

**TABLE 3:** “Input Seqs” denotes the number of input sequences for each batch in the second iteration; “Sub-clusters” denotes the number of sub-clusters obtained from each batch; “Singletons” denotes the number of singletons obtained for each batch; “Output Seqs” gives the number of sequences to be used in the third iteration; and “Out/In” indicates the reduction in size of the sequences to be used in the third iteration compared to the second. The reduction in input size for the third iteration is almost 30%. Each value in the “Output Seqs” column is the sum of the number of sub-clusters and the number of singletons. The first sequence in each sub-cluster was used to represent the entire sub-cluster in the third iteration.

Iter 2	Input seqs	Sub-clusters	Singletons	Output seqs	Out/In (%)
Batch_123_	653,218	110,985	375,672	486,657	74.50
Batch_4567_	752,255	142,130	333,160	475,290	63.18
Batch_891011_	641,290	98,555	397,303	495,858	77.32
Total	2,046,763	351,670	1,106,135	1,457,805	71.22

For the third iteration, we repeated the same steps as used for the first two except that duplicate sequences were removed, and only Batch_123 and Batch_4567 were combined and used as the input to pClust. Values are presented in Table 4.

**TABLE 4:** “Input Seqs” denotes the number of input sequences for each batch in the third iteration; “Sub-clusters” denotes the number of sub-clusters obtained from each batch; “Singletons” denotes the number of singletons obtained for each batch; “Output Seqs” gives the number of sequences to be used in the fourth iteration; and “Out/In” indicates the reduction in size of the sequences used in the fourth iteration compared to the third. The reduction in input size for the fourth iteration is about 22%. Each value in the “Output Seqs” column is the sum of the number of sub-clusters and the number of singletons. The first sequence in each sub-cluster was used to represent the entire sub-cluster in the fourth and final iteration.

Iter 3	Input seqs	Sub-clusters	Singletons	Output seqs	Out/In (%)
Batch_1234567_	960,807	170,279	595,586	765,865	79.71
Batch_891011_	641,290	98,555	397,303	495,858	77.32
Total	1,602,097	268,834	992,889	1,261,723	78.75

There were 1,261,723 output sequences at the conclusion of the third iteration, but after removal of duplicate sequences, 1,260,580 were retained and used as the input to pClust for the fourth and final iteration. The number of input sequences and sub-clusters for the final iteration are presented in Table 5.

**TABLE 5:** “Input Seqs” denotes the number of input sequences in the single batch in the fourth and final iteration; “Sub-clusters” denotes the number of sub-clusters obtained.

Iter 4	Input seqs	Sub-clusters
Batch_1234567891011_	1,260,580	194,521

#### 2.1.4 Sub-Cluster Merging

After completing the iterative clustering, we proceeded to sub-cluster merging. We start with the sub-clusters found in the fourth and final iteration. The basic idea is to map the sub-clusters and singletons to the results of the third iteration, then to the results of the second iteration, and finally to the results of the first iteration, merging all the sequences to create homologous clusters. This is possible because of the naming convention we adopted as described previously. Briefly, sequences from different batches are identified by concatenating the sub-cluster name and sequence index with the batch name.

**FIGURE 1:**
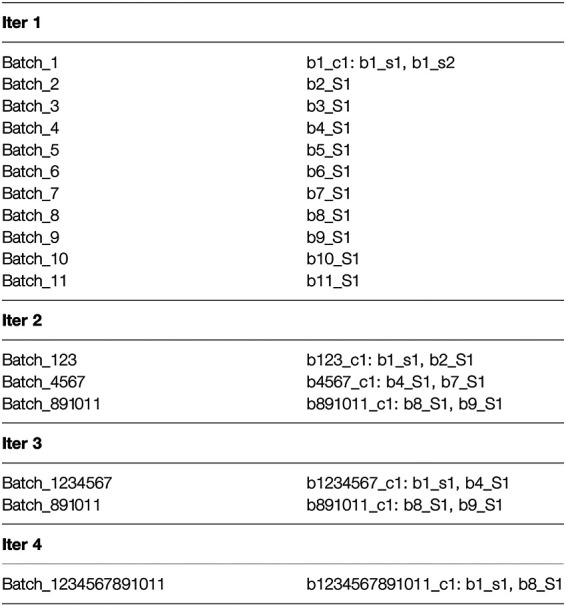
We illustrate the sub-cluster merging process using a toy example. This example doesn't represent realistic results and doesn't use our naming convention. The simplification is meant to provide an understanding of the concept underlying the formation of our final clusters. This figure presents the partial results after completion of the iterative clustering step.

**FIGURE 2:**
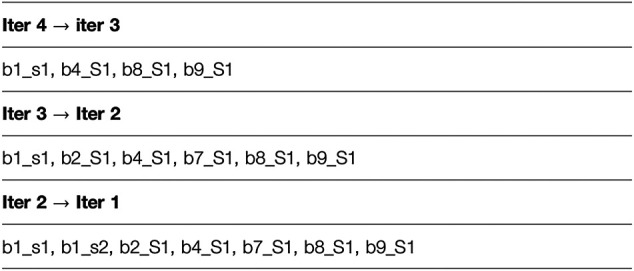
We illustrate the sub-cluster merging process using a toy example. This example doesn't represent realistic results and doesn't use our naming convention. The simplification is meant to provide an understanding of the concept underlying the formation of our final clusters. This figure presents the results after mapping and merging.

Next, we illustrate the sub-cluster merging process using a toy example. This example doesn’t represent realistic results and doesn’t use our naming convention. The simplification is meant to provide an understanding of the concept underlying the formation of our final clusters. After completion of the iterative clustering step, we assume the partial results shown in Figure 1, where “bi” indicates batch number “i”, “cj” indicates cluster number “j”, “sk” indicates sequence number “k”, and “Sm” indicates singleton number “m”. As shown, the results of the first iteration are a single cluster in Batch_1 comprised of two sequences b1_s1 and b1_s2 and one singleton each in Batch_2 through Batch_11. For the second iteration, only the first sequence b1_s1 is used to represent the cluster b1_c1, and this sequence clusters with the singleton b2_S2 to form the cluster b123_c1. In addition, the second iteration results in two more clusters composed of singletons from the first iteration. For the third iteration, again only single sequences from each of the clusters b123_c1 and b4567_c1 are used to represent them (note that b891011_c1 is not used for this iteration), and the third iteration results in the two clusters as shown (with b891011_c1 retained from the second iteration). Finally, the fourth iteration results in a single cluster b1234567891011_c1 comprised of the two single representative sequences b1_s1 and b8_S1 from the previous iteration. Then mapping and merging give the results shown in Figure 2. We see how the cluster membership expands as we move from the last iteration to the first. For example, the two sequences in cluster b1234567891011_c1 from the last iteration are mapped and merged with sequences from the third iteration. The final result is a single homologous cluster with sequences b1_s1, b1_s2, b2_S1, b4_S1, b7_s1, b8_S1, and b9_S1 and the five singletons from the first iteration that didn’t cluster (b3_S1, b5_S1, b6_S1, b10_S1, and b11_S1).

With more than nine million protein sequences, the algorithm used to accomplish the sub-cluster merging step was non-trivial. The end result was 361,135 homologous clusters ranging in size from 2 to 26,577 sequences, an average of 23 sequences, and a median of 4 sequences (numerous clusters have only 2 sequences), as well as 818,791 singletons. The membership information for each of the ∼9.26 million sequences populating the clusters and singletons consists of its identification number, accession number, annotation, cluster identification, and a validation flag denoting whether its function has been experimentally validated. We store this membership information as well as the mapping of an organism to its corresponding order in a PostgreSQL relational database (Stonebraker and Kemnitz, 1991), which is a free and open-source object-relational database management system that can handle the workload required for large web applications with many concurrent users. The FASTA files for all the sequences are integrated into a BLAST database.

### 2.2 An Automated, Smart Natural Language Processing Tool

To determine whether functional annotation has been confirmed for a protein sequence, we developed a smart natural language processing (NLP) tool which periodically and automatically queries the UniprotKB database to determine whether a protein function has been experimentally verified. The input to our smart program are publication titles retrieved from the UniProtKB database that are linked to a given accession number for a protein sequence; the output is a binary prediction of whether protein function has been experimentally verified. Prediction uses the results of recurrent convolutional neural network, logistic regression, and support vector machine models and employs a voting scheme. The NLP is based on word embeddings and uses BioWordVec (Zhang et al., 2019) and BioSentVec (Chen et al., 2019). Complete details are given in (Tao et al., 2021).

Our smart program is integrated into the PASS website as a back-end program which is inaccessible to PASS users. It is executed automatically and periodically based on an estimate of when a sufficient amount of new data are available in the UniprotKB database. This estimate can be changed. During program execution, each accession number for a sequence yet to be validated is mapped to the entry identifier in the UniprotKB database. If the entry is in the UniProtKB/Swiss-Prot database, which is the manually curated section of the UniprotKB database, the program checks for publications categorized as a “Function” publication. If such a publication exists, a validation flag is changed from 0 to 1 in the PASS PostgreSQL database. If the entry is in the UniProtKB/TrEMBL database, publication titles associated with the entry are extracted and used as input to the ML tool. If at least one publication associated with a protein entry is classified as positive, the publication title(s) and the protein accession number are submitted to a human curator for manual review (Tao et al., 2021). The curator is responsible for changing the validation flag after reviewing the relevant publication(s). Sequences with verified functional annotation are identified by FASTA headers in red font.

At the time of writing, our smart program had identified 370 protein sequences in the UniProtKB/Swiss-Prot database whose function has been experimentally validated. Initial use of the NLP algorithm identified 814 protein sequences with publications potentially containing proof of experimental validation. These publications are currently awaiting review by our human curator. Subsequent deployment of the NLP algorithm will likely result in significantly smaller numbers. The total number of sequences, less than 1200, is a small fraction of the total number of sequences in the database, but this number will increase with time. In the meantime, sequences with consensus annotations will provide useful guidance for the correct annotation, and when there is no clear consensus, the SSN, described in the next section, will allow a user to determine the annotation most likely to be correct for their sequence.

### 2.3 Sequence Similarity Networks

A sequence query submitted by a PASS user will return a list of FASTA headers for the cluster that best matches the query. When there is a consensus annotation, the user will have no issue, but if there are distinctly different annotations, the user will have to decide which annotation to use. The user can either infer the correct annotation based on their expertise or else create a sequence similarity network (SSN) on our website. Because homology does not guarantee the same molecular function, an SSN can assist a user with choosing an accurate annotation. The user simply selects a minimum of two sequences from the FASTA headers in the cluster, including any that have been verified, and an SSN is generated. Visualization of the SSN is implemented by NetworkX, a Python package for network analysis, based on the pairwise distances between sequences provided in a distance matrix. The best match in the SSN is listed, where 0 is a perfect match, i.e., sequences are identical.

To create the distance matrix, we use Clustal Omega (Sievers et al., 2011). Clustal Omega provides a fast and accurate alignment program and generation of the pairwise distance matrix used in NetworkX to create the SSN. To create the distance matrix, Clustal Omega compares unaligned sequences using *k*-mer distance (Wilbur and Lipman, 1983).

### 2.4 The Protein Annotation Surveillance Site Query-Able Website

**FIGURE 3:**
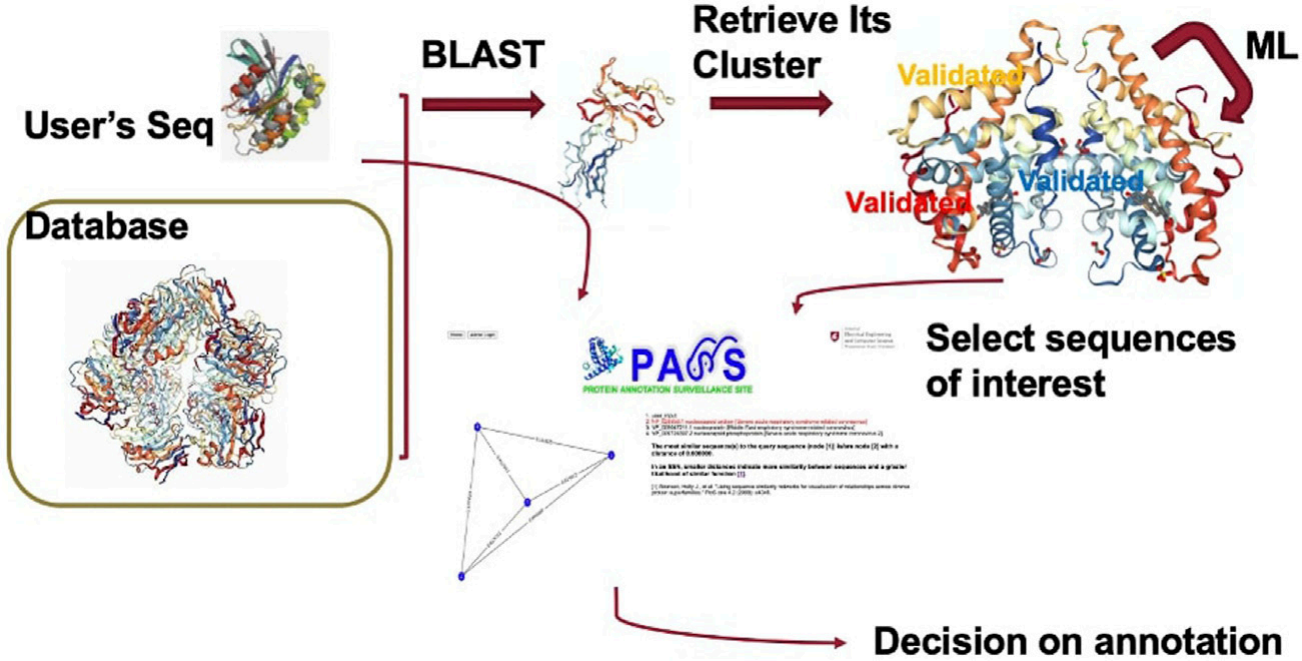
PASS flowchart demonstrating the query process.

**FIGURE 4:**
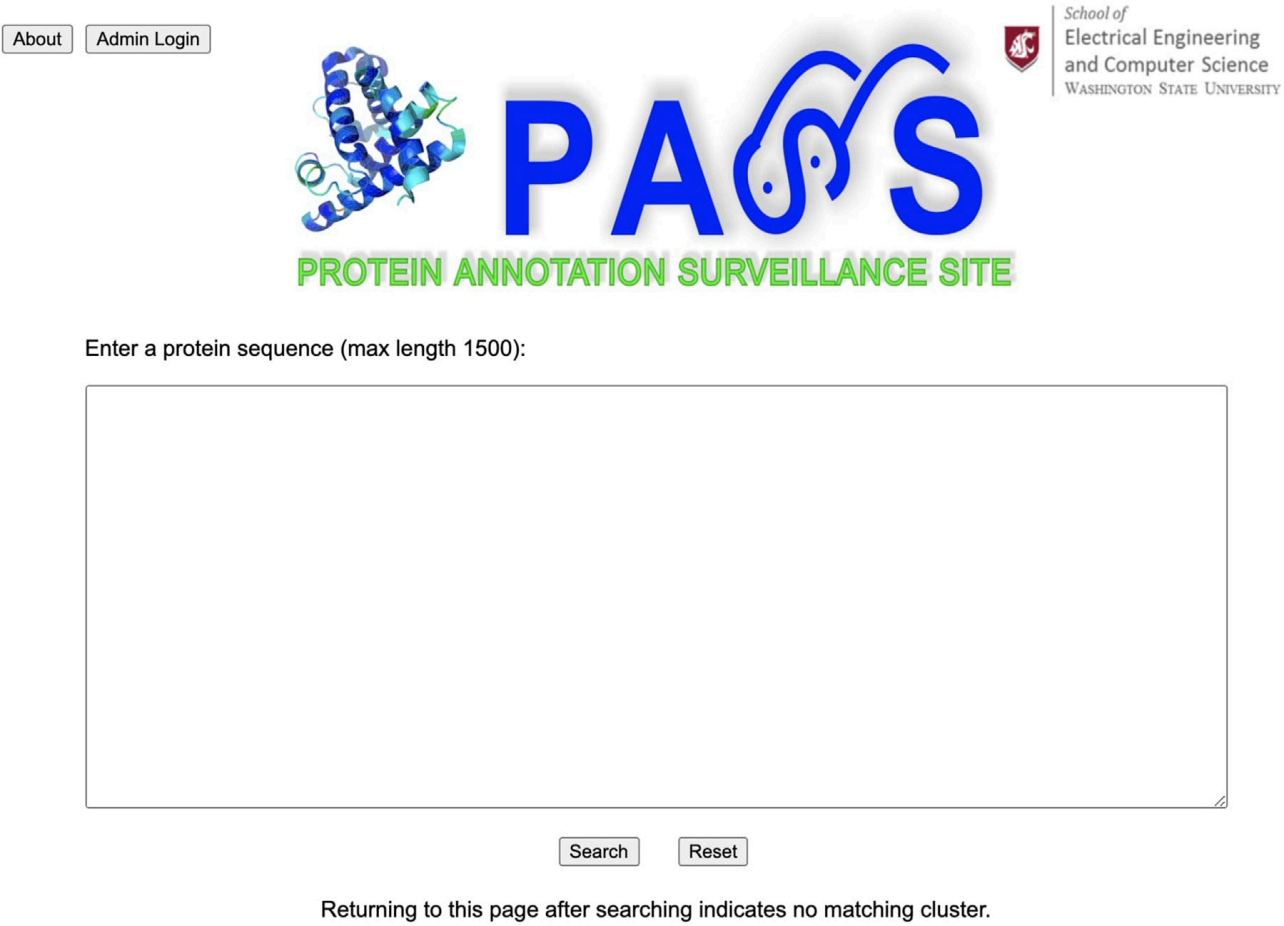
PASS home page showing the query window where a user enters their protein sequence.

The Protein Annotation Surveillance Site (PASS) is implemented in a clear and easy-to-use style using Django, a free, open-source web framework which uses Python. The PASS flowchart is provided in Figure 3. The web page interface links directly with the underlying database system. Figure 4 displays the PASS homepage with the query window. Administration of the PASS website is performed directly from a web browser for sequence curation and via the host server for other functions.

After a user enters their query sequence and clicks on the search button, the query engine looks for similar sequences using BLAST and retrieves the cluster with the best match, displaying the header of the FASTA file with annotation information for each sequence in the cluster. If the query is unsuccessful, the user is returned to the homepage. The user can scroll through the list of all cluster members and select the sequences they believe are most likely to be related to their query sequence to create an SSN. Any sequences that have been validated experimentally are highlighted in red. When available, the user can include sequences of proteins that have been experimentally validated to make a more informed annotation choice. In the next section we present several examples to demonstrate use of the PASS website.

## 3 Results and Discussion

In this section, we present two use cases to demonstrate the use of PASS. We consider two protein sequences, one from the yeast *Kluyveromyces lactis* and one from a coronavirus.

### 3.1 Example 1: Eukarya

**FIGURE 5:**
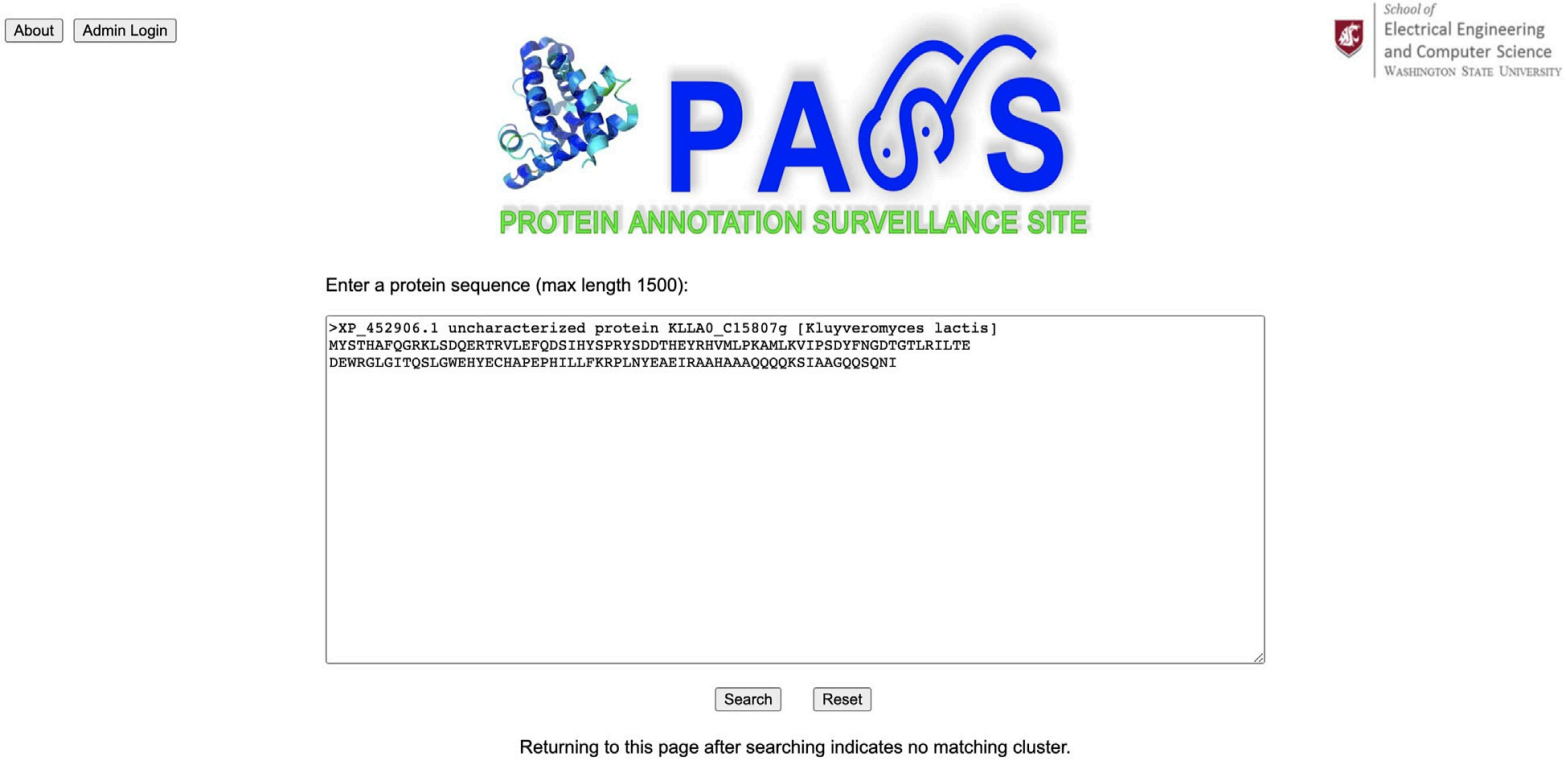
The protein sequence annotated as uncharacterized protein KLLA0_C15807 g from the yeast *Kluyveromyces lactis* is used as the query sequence in PASS.

We use the protein sequence annotated as uncharacterized protein KLLA0_C15807 g from the yeast *Kluyveromyces lactis* as the query sequence for the PASS website as shown in Figure 5. Its complete FASTA file is available at www.ncbi.nlm.nih.gov/protein/XP_452906.1?report=fasta. The FASTA file can be used with or without its header.

**FIGURE 6:**
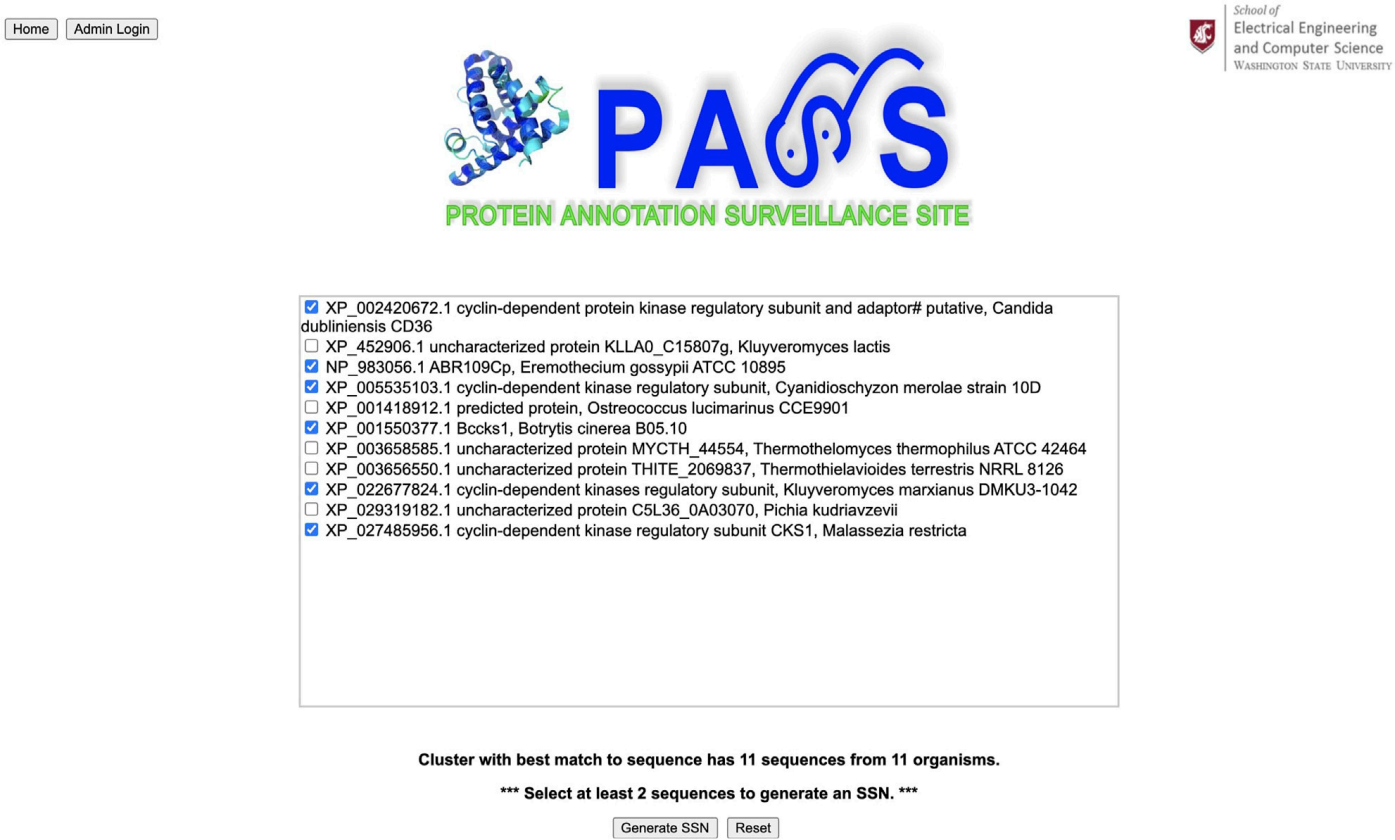
The query sequence for the yeast protein matches a cluster with eleven sequences, and the six sequences with annotations are chosen to create a sequence similarity network (SSN).

**FIGURE 7:**
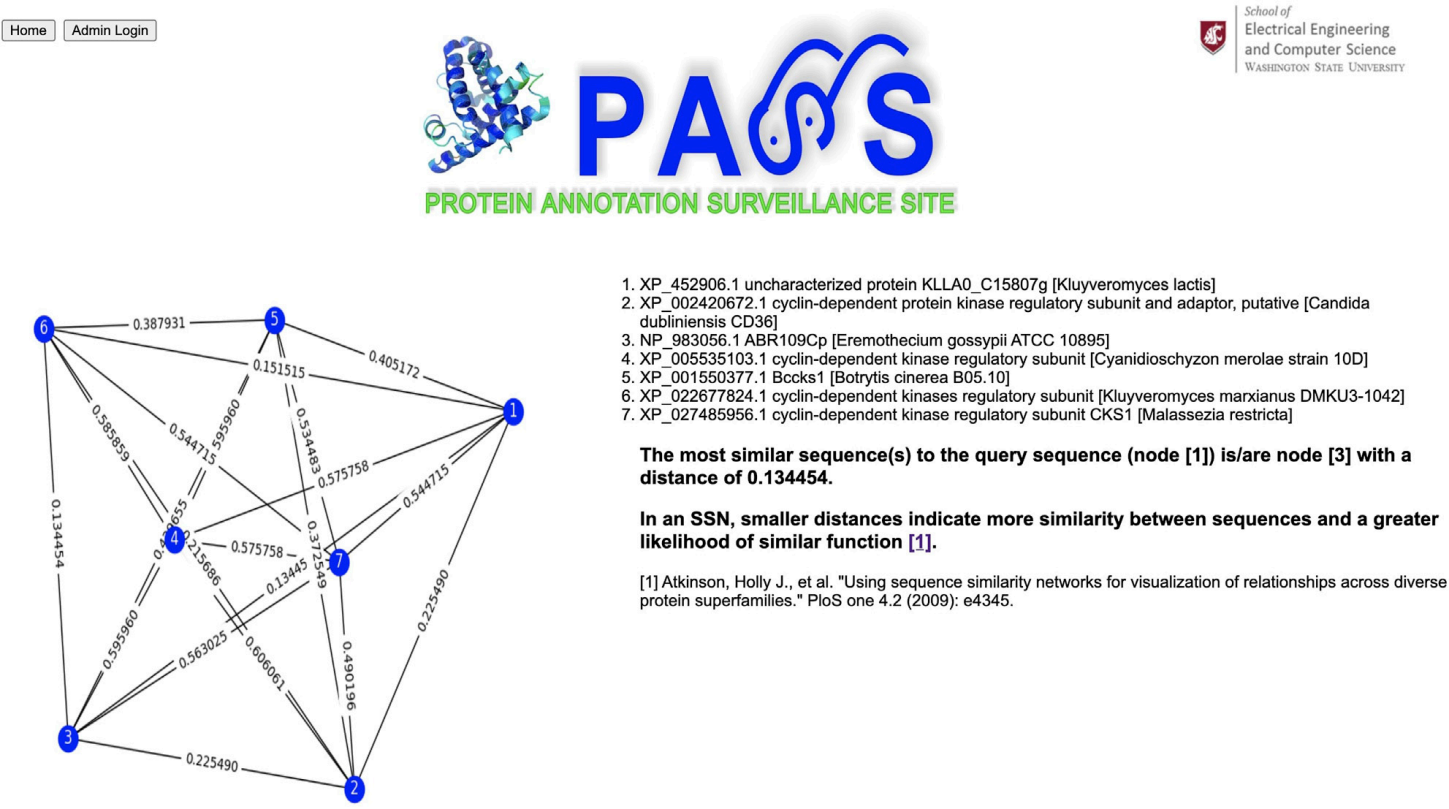
Sequence similarity network (SSN) composed of the query sequence and sequences selected by the user. In an SSN, smaller distances indicate more similarity between sequences and a greater likelihood of similar function.

Our query sequence matches a homologous cluster containing eleven sequences (Figure 6). We choose the six sequences with annotations to create a sequence similarity network; the input sequence is automatically included in the SSN. From the SSN in Figure 7 we note that the query sequence is most similar to the sequence with accession number NP_983056.1, with a distance of 0.134454, and annotated as ABR109Cp. However, this annotation is not particularly informative, and the majority of sequences are annotated as cyclin-dependent kinase regulatory subunit. In addition, the distance between, for example, our query sequence and sequence #6, annotated as cyclin-dependent kinase regulatory subunit, is 0.151515 which indicates close similarity between them as well. Thus, it is probably best to use the majority annotation in this example.

### 3.2 Example 2: Virus

**FIGURE 8:**
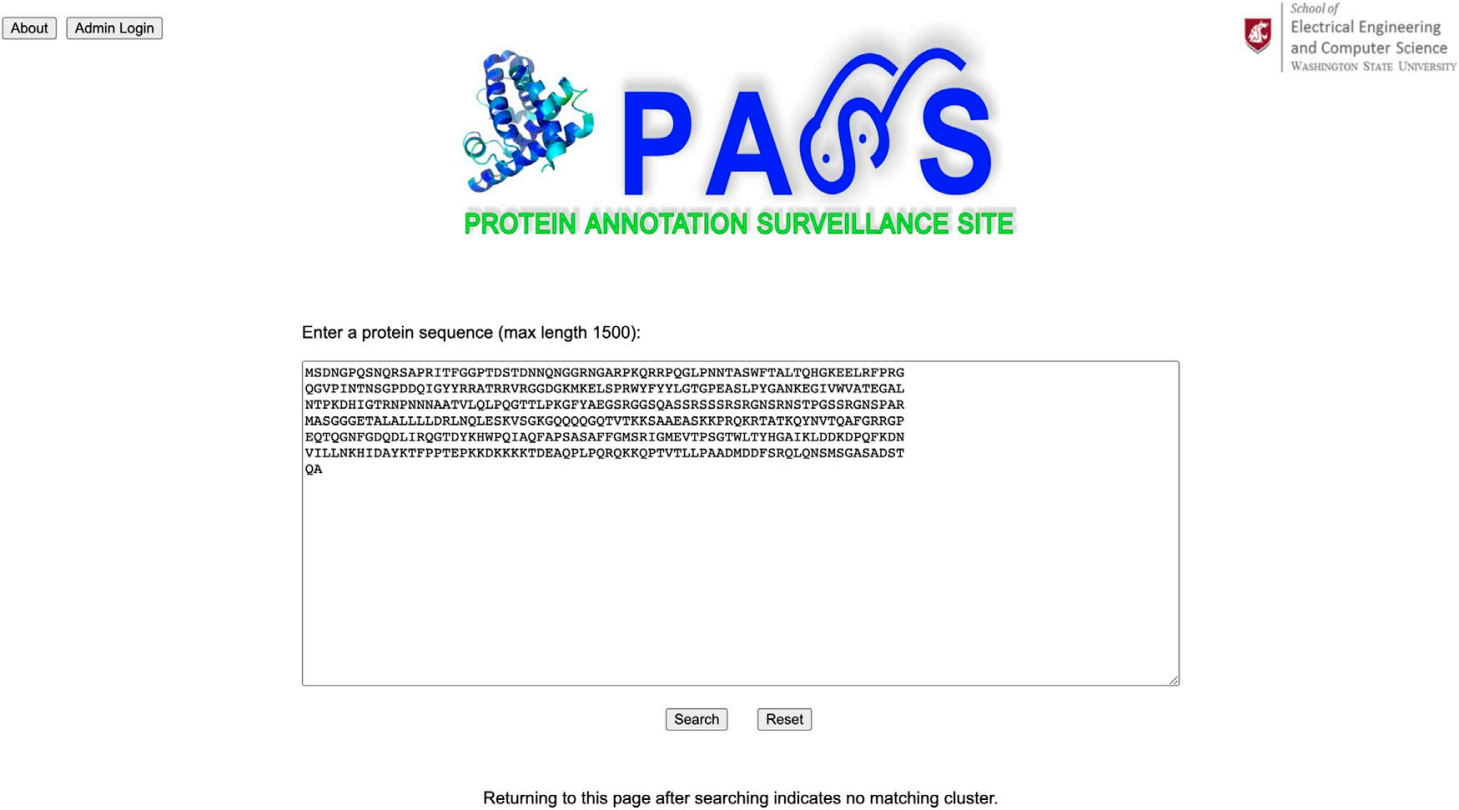
A spike protein from a coronavirus (CoV) is used as the query sequence in PASS.

**FIGURE 9:**
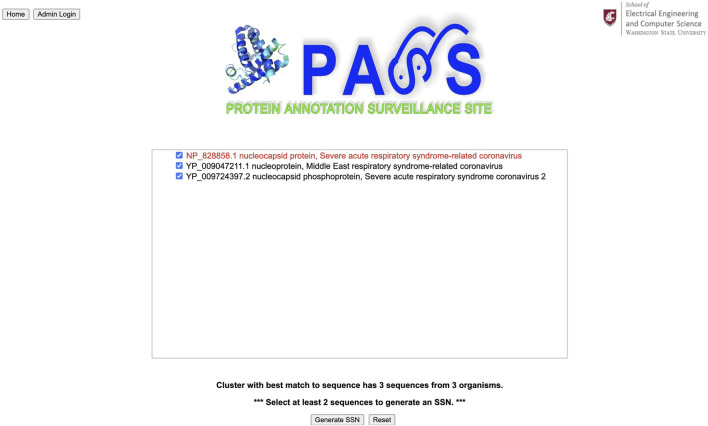
The query sequence for the coronavirus spike protein matches a cluster with three sequences. All three sequences are chosen to create an SSN.

**FIGURE 10:**
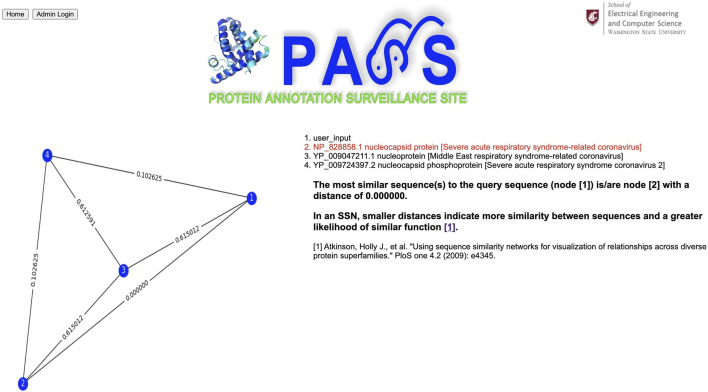
Sequence similarity network (SSN) composed of the query sequence and sequences selected by the user. In an SSN, smaller distances indicate more similarity between sequences and a greater likelihood of similar function.

For our second example, we use a spike protein from a coronavirus (CoV) as our query sequence as shown in Figure 8; its complete FASTA file is available at www.ncbi.nlm.nih.gov/protein/YP_009825061.1?report=fasta. The query sequence matches a homologous cluster with three sequences as shown in Figure 9. One of the three cluster sequences is highlighted by red font, indicating that the function of this sequence has been experimentally validated. We choose all three sequences to generate the SSN shown in Figure 10 where we see that the query sequence is identical to the validated sequence (#2), i.e., the distance between the two sequences is 0, with accession number NP_828858.1 and annotated as nucleocapsid protein from SARS-CoV. Interestingly, sequence #2 is from SARS-CoV, sequence #3 is from MERS-CoV, and sequence #4 is from SARS-CoV-2. From the SSN results, it’s most likely that the query sequence is from SARS-CoV. The SSN also shows that the nucleocapsid proteins for SARS-CoV and SARS-CoV-2 are more similar to each other than to the nucleocapsid protein of MERS-CoV.

## 4 Conclusion

In this work, we presented the Protein Annotation Surveillance Site (PASS), a system developed to help life scientists with protein annotation and their fundamental research on proteins. The proposed system has multiple advantages. First, our database contains homologous clusters of close to 8.5 million protein sequences deduced from the representative genomes of bacteria, archaea, eukarya, and viruses. Our data source for protein sequences is the National Center for Biotechnology (NCBI) Reference Sequence (RefSeq) collection which contains a comprehensive, non-redundant, and well-annotated set of protein sequences. Our system provides a foundation for protein-related studies, and protein sequences in our database have the potential to serve as useful references for sequences of interest to users. Second, we developed an innovative approach for clustering millions of protein sequences into homologous groups using a two-step approach. The first step involved iterative clustering. Batches of sequences were clustered, results from each batch were used to create smaller batches, and this process was repeated until sequences from a single batch were clustered. In the second step, results from the final iteration were merged with the previous iteration, and the procedure continued until merging of the initial results was completed. Our two-step approach is efficient, and it overcomes the constraints of limited computational power. Third, our system uses peer-reviewed publications with experimental validation of protein function. Because of the dearth of existing experimental verification, extension of this information from individual protein sequences to other protein sequences is valuable, and our system provides this capability. In addition, given that homology does not guarantee the same molecular function, the ability to create sequence similarity networks for sequences of interest to a user, which is provided by our system, assists in preventing assignment of annotation based only on homology. Altogether, use of our PASS system website assists users with the accurate annotation of protein sequences of interest to them. Finally, we developed a novel ensemble machine-learning (ML) program that uses natural language processing via word embeddings and voting based on the results of three different models. Our ML algorithm has been integrated into the PASS system and automatically provides updates from the UniProtKB database. Function-related publications in the curated UniProtKB/Swiss-Prot database are used for direct validation of protein function for sequences in our PASS database, while publications in the uncurated UniProtKB/TrEMBL database are used with our ML program, and if prediction is positive, the results are passed to a human curator for manual review to guarantee 100% validation accuracy.

Several enhancements to the PASS system are possible. First, the incorporation of active learning to our ML program is likely to improve its current performance. When a publication that doesn’t provide experimental evidence of protein function is incorrectly predicted to provide this evidence, the error will be caught by our human expert. After a sufficient number of errors have been found, these data can be combined with the existing training data to retrain the model. Second, the number of complete representative genomes will continue its exponential growth. While it’s probable that the majority of protein sequences for bacteria already exist in the database, this is not true for archaea, eukarya, and viruses simply because the majority of genomes in the NCBI database are bacterial. Protein sequences deduced from the genomes of archaea, eukarya, and viruses can be clustered as they become available, and then BLAST can be used to determine whether the clusters or singletons match clusters already in the database. If so, they can be added to these clusters; if not, they can be added to the database as new clusters. Third, an interesting direction would be to implement the two-step approach of iterative clustering and sub-cluster merging in parallel. This would help to eliminate the constraints of computational resources and make homologous clustering at any scale possible.

## Data Availability

The Protein Annotation Surveillance Site (PASS) can be accessed at https://pass.eecs.wsu.edu/.
